# Multi-Component Physical Activity Interventions in the UK Must Consider Determinants of Activity to Increase Effectiveness

**DOI:** 10.3390/jfmk6030056

**Published:** 2021-06-23

**Authors:** Mark A. Faghy, Kirsty E. Armstrong-Booth, Vicki Staples, Micheal J. Duncan, Clare M. P. Roscoe

**Affiliations:** 1 Human Sciences Research Centre, University of Derby, Derby DE22 1GB, UK; k.armstrongbooth1@unimail.derby.ac.uk (K.E.A.-B.); c.roscoe@derby.ac.uk (C.M.P.R.); 2 School of Psychology, University of Derby, Derby DE22 1GB, UK; v.staples@derby.ac.uk; 3 Department of Applied Sciences and Health, Coventry University, Coventry CV1 5FB, UK; aa8396@coventry.ac.uk

**Keywords:** physical activity, sedentary behaviour, theoretical approaches, behaviour

## Abstract

Interventions to increase physical activity in children have adopted broad approaches and achieved varying success. There is a need to adopt approaches underpinned with a theoretical basis. Accordingly, the aim here was to implement and evaluate a 12-week intervention designed using the concepts of the COM-B model to determine the effect this has on physical activity levels. One hundred and forty-seven school-age children (mean age 8.9 ± 1.3 years) took part in a 12-week program delivered in a school setting. Topics included physical activity, healthy eating, sleep quality and reducing screen time/sedentary activities when not in school. A sample of participants wore a wrist-worn accelerometer for seven days pre-and post-intervention (N = 11). The physical activity frequency was unchanged (2.9 ± 1.0 AU) when compared with post-intervention values (3.1 ± 0.8 AU, mean increase 6.8 ± 3.7%, *p* > 0.05). Changes were observed in the daily consumption of fruit and vegetables (pre-intervention 44.6% vs. post-intervention 60.2%, *p* < 0.05). Sedentary time, light activity, moderate activity and vigorous activity were unchanged post-intervention (*p* > 0.05). There is a need to adopt a broader approach that incorporates a theoretical basis and considers the complex ways by which physical activity behaviours are influenced.

## 1. Introduction

Despite the well-documented benefits of regular physical activity in children, there has been a prominent rise in the levels of inactivity with statistics demonstrating that 79% of boys and 84% of girls aged between 5–15 years do not achieve the recommended levels of physical activity (an average of at least 60 min of moderate-to-vigorous activity per day) [1]. Data also highlight throughout childhood and into adolescence that levels of physical activity continue to fall [2]. Concurrently, and a known determinant of the reduction in physical activity, is the rise in sedentary behaviours outside of school as recent data suggest that children are most active during school hours [3] with 43% of boys and 37% of girls aged 13–15 years achieving >6 h of physical activity at a weekend [4]. This after-school time window is important in maximising opportunities to be physically active but review data suggest that children are spending more time undertaking sedentary behaviours such as watching TV, other screen-based activities, motorised transport and homework during this period [5]. 

Reductions in physical activity levels and increased sedentary time have contributed to the current increased childhood obesity levels, which is considered by the World Health Organisation to be one of the most serious public health challenges of the 21st century [6] and with serious epidemiological and economic consequences [7]. In recognition of this, there is a need to develop large scale preventative strategies that seek to increase physical activity levels and reduce sedentary time in children. This is important due to the broad implications to population health not only in children and young adults but also across the lifespan [8] as promoting active lifestyles during childhood is crucial as childhood physical activity levels track into adulthood with the potential to provide widespread public health benefits [9].

To date, school-based interventions with children have been conducted, adopting a wide range of approaches in both the design and implementation [10,11]. Largely, the development of interventions has centred solely on increasing physical activity levels and the efficacy of such interventions has been reviewed with varying success, which has questioned the methods and measures and led to recommendations for future interventions [12,13]. Howlet et al. [14] report that only 56% of interventions apply theoretical concepts in their design and only 24% of these measure pre- to post-intervention changes. These recommendations suggest that interventions must be at least 12 weeks long to promote meaningful change; however, of the published interventions there is a variability in the length ranging from four to eight weeks. The need to adopt a multi-component approach that tackles the broader determinants of physical activity including reducing sedentary behaviour alongside tackling contemporary issues around excessive screen time, sleep and healthy food choices has also been highlighted [15,16]. Such assertions are also supported by a strong theoretical underpinning [17]. The COM-B model (capability, opportunity and motivation behaviour) is a framework with clear links to specific behaviour change techniques as described in the Behaviour Change Wheel and Taxonomy of Behaviour Change Techniques [18], which is particularly applicable for the design and evaluation of multi-component interventions. The model offers an opportunity to design and implement interventions and in understanding and assessing existing interventions mapped against the framework. The components within the COM-B model focus on capability, opportunity and motivation as factors to predict and reflect behaviour; however, there is limited evidence that demonstrates the effectiveness of this model on creating long-lasting change in physical activity levels. There is a need to develop and evaluate interventions that consider the broader determinants and influencers of physical activity to address the increasing prevalence of the sedentary behaviours of school children. Accordingly, the current study aims to investigate changes in physical activity (primary outcome) and the known determinants of physical activity (sedentary behaviours, use of technologies, healthy eating, sleep and screen time) following a 12-week multi-component school-based intervention designed using the theoretical basis and concepts of the COM-B model. 

## 2. Materials and Methods

### 2.1. Participants

Following an ethics approval from the University’s Human Science Ethics committee (AB-AUT-01-VS), one hundred and forty-seven (seventy-four males) took part in the ‘Physically Active Children of Erewash’ (PACE) program over 12 months. Consent was obtained from individual schools and parents of each child that entered the program. All participants attended primary and secondary schools within the Erewash district of Derbyshire with six schools taking part in total. All children were aged between 7 and 11 years (key stage 2–3, mean age 8.9 ± 1.3 years) and were grouped according to the school year and in groups of approximately 10–12 children. 

### 2.2. Procedure

All participants were asked to complete a baseline questionnaire that was made available via an online platform (Qualtrics TM) and via a printed version. The participants completed this process independently but could ask for guidance if they required the questionnaire to be read to them or if any questions needed explaining. The components of the questionnaire are detailed below and were collected before and after a 12-week intervention (also detailed below). Participation in the program was voluntary and each participant received a participant pack following the completion of the baseline data sets, which contained a pedometer, water bottle and sports bag for their participation in the program. Upon completion of the intervention, the participants completed the same questionnaire as at before; however, the post-intervention questionnaire also contained a process evaluation to assess and capture free-text responses relating to enjoyment, satisfaction and learning of the 12-week intervention. 

In addition, and to provide an objective assessment of physical activity and sleep, a sample of participants (*N* = 11) was recruited to wear a wrist-worn GENEActiv Original accelerometer (ActivInsights Ltd., Kimbolton, Cambridgeshire, UK) attached using a watch strap and positioned over the dorsal aspect of their wrist midway between the radial and ulnar styloid process on their non-dominant hand for seven days, following baseline data collection and again before the end of the intervention, adopting the protocol of Dillon et al. [19]. Accelerometers worn on the wrist are more convenient to wear and lead to greater compliance during prolonged wearing when assessing habitual activity [20]. During this time, participants were instructed to wear the device continuously. The GENEActiv is a lightweight triaxial accelerometer that provides raw acceleration data and has previously been described in detail [21]. The device has high intra- and inter-instrument reliability (coefficient of variation = 1.8% and 2.4%, respectively), good criterion-referenced validity (r = 0.97) [21] and has been tested for reliability and validity in both adult and children populations [22,23,24]. 

### 2.3. Measures

The questionnaire was designed and tested via pilot work to confirm age appropriateness and it was considered suitable for all of the age groups taking part. All questions were mapped onto both the COM-B model [18] to assess the capability, opportunity and motivation and the Theoretical Domains Framework (TDF) [25], which were designed to test the components of the intervention (described below). Firstly, participants answered questions about themselves before completing four sections (physical activity, food and drink, sleep and screen time) that corresponded with the topics covered in the content of the intervention. All questions used a 4-point Likert scale and included items such as “I do more than 60 min (1 h) of activity per day”. The children then had to choose between four possible answers; for example, every day, most days, some days and no days. Interactive sliding scales and smiley faces were also included to engage the target population and ensure questionnaires were completed fully and interest was not lost. The post-intervention questionnaire also included further questions about how the children found the PACE program, what they had learnt (knowledge assessment) and if they would recommend it to others. 

Accelerometer data were sampled at 100 Hz at a time interval (epoch) of 60 s and were reported as counts per minute (CPM). Following the return of the accelerometer, data were analysed from the three planes of motion and collapsed using the sum of vector magnitude equation (SVMgs; $\sum \sqrt{\left(x^{2}+y^{2}+z^{2}\right)}-g$. Each period was categorised based on the value of the SVMgs with specific cut-points used to determine the intensity of the physical activity achieved. Non-dominant cut-offs were <158.5 SVMgs (sedentary), <261.8 SVMgs (light), <465 SVMgs (moderate) and ≥465 SVMgs (vigorous), as detailed previously by Mackintosh et al. [26]. 

### 2.4. Intervention

The intervention was a multi-component education and activity program developed to address a range of health behaviours and physical activity, which was delivered during term-time within a school setting using an established ‘Feel Good Factor’ toolkit. The toolkit was devised in partnership with the Bolsover and North-East Derbyshire commissioning partnership to tackle inactivity and weight issues with young people. The toolkit takes a universal approach using a collection of ideas and activities, aiming to increase skills and confidence and delivering consistent health messages in a fun, engaging and interactive way. The intervention was delivered over 12 weeks to schools in the East Derbyshire (UK) catchment area. The sessions were delivered face to face by the researchers and teachers and lasted 60 min and were delivered weekly during school time and in a school/classroom setting. Each week, different content and topics were covered with activity sessions and information materials (activity sheets, traffic light evaluations) that incorporated several educational (information sheets) and interactive tasks (games and quizzes). The topics covered in the intervention included physical activity, healthy eating, sleep quality and reducing screen time/sedentary activities when not in school. The topics covered were consistent across all schools but were tailored to ensure the appropriateness of the different age groups. 

### 2.5. Statistical Analysis

The Likert scores were treated as interval data for an analysis and reported as arbitrary units (AU) and pre- to post-intervention changes were assessed with a series of independent and dependent t-tests. Descriptive statistics were calculated for all variables; all values are reported as mean ± SD. The normal distribution was assessed through a visual inspection of the frequency histograms and a Shapiro–Wilk test and all parametric assumptions were satisfied. Pre- to post-intervention changes in GENEActiv and subsequent domains were analysed using paired t-tests to determine pre- and post-intervention differences in physical activity levels and intensity profiles. All data are presented as mean ± SD with an alpha level of <0.05 used to denote statistically significant differences. All data were analysed using IBM SPSS Statistics (version 24, Armonk, NY, USA). 

## 3. Results

### 3.1. Physical Activity

Engagement with physical activity was unchanged from pre-intervention values (2.9 ± 1.0 AU) when compared with post-intervention values (3.1 ± 0.8 AU) with 26% (*N* = 39) of participants reporting an increase in the number of days when they completed more than 60 min of exercise (*p* > 0.05). When compared for gender, there were no differences in the frequency completed by males and females at either the baseline (males 2.9 ± 1.0 and females 2.9 ± 0.9, *p* < 0.05) or post-intervention (males 3.1 ± 1.0 and females 3.2 ± 0.7, *p* < 0.05). There was also an increase in the reported knowledge of being active regularly with 55% (*N* = 81) of participants reporting that they thought it was important to be active each day at the baseline, which was improved post-intervention (83.8%, *p* < 0.05). The participants reported that they were more likely to be active with their family (60.0%) than with their friends (48.4%, *N* = 71) at the baseline; post-intervention, there was an increase in engaging with activity with their friends (69.2%, *N* = 101, *p* < 0.05) but being active with family members was unchanged (69.5%, *p* > 0.05). 

### 3.2. Food and Drink

A total of 44.6% (*N* = 66) of participants reported eating fruit and vegetables daily, which increased to 60.2% (*N* = 88) post-intervention (*p* < 0.05) and was similar between genders at both timepoints (males 3.4 ± 0.9 AU and females, 3.6 ± 1.1 AU, *p* > 0.05). At the baseline, the number of participants reporting that they enjoyed consuming healthy foods and snacks was 48.1% (*N* = 71) and this was similar following the intervention (46.6%, *N* = 69, *p* > 0.05). A high number of participants reported consuming fizzy drinks (pre: 9.4% vs. post: 10.2%), sugary foods (pre: 17.3% vs. post: 15.6%) and salty foods (pre: 10.2% vs. post: 10.3%) at the baseline, which was unchanged in all categories following the intervention (*p* > 0.05). The participants that reported eating meals at a table with their family was 51.6% (*N* = 76) at the baseline and increased following the intervention (74.8%, *N* = 110, *p* < 0.05). Similarly, 16.5% (*N* = 24) of participants reported that they ate their meals in front of the television and this was similar following the intervention (13.3%, *p* > 0.05). 

### 3.3. Sleep

The participants reported an average bedtime on a school night of 19:47 ± 1:32 h and a wake-up time of 07:30 ± 0:48 min at the baseline, which was unchanged following the intervention, with an average sleep time of 9.5 ± 1.6 h. The participants reported that they slept well and woke up feeling good most mornings (27.5%, *N* = 40), which was unchanged post-intervention (28.1%, *p* > 0.05). Conversely, 26.7% (*N* = 39) of the participants reported that they felt tired in the mornings, which was unchanged following the intervention (24.2%, *p* > 0.05). More than half of the participants reported playing a game console or watching TV directly before they went to bed every night or most nights, which was unchanged following the intervention (pre: 55.5% vs. post: 56.2%, *p* > 0.05). The number of participants that reported reading or being read a story before bedtime increased from 14.1% (*N* = 21) to 28.3% (*N* = 42, *p* < 0.05) following the intervention. 

### 3.4. Screen Time

The mean access to digital platforms was 4.6 ± 2.1 devices per child and this included access to iPads, games consoles, mobile phones, smartwatches and laptop and desktop computers. The frequency of using electronic devices for more than one hour per day was similar following the intervention (pre: 53.3% vs. post: 57.9%, *p* > 0.05). A high percentage of participants also reported using a screen before leaving for school (36.4%, *N* = 54) and whilst eating meals (27.1%), which was unchanged following the intervention (35.8% and 28.8%, respectively, *p* > 0.05). The number of participants that reported being able to use electronic devices without a time limit was also high (30.7%, *N* = 45) and this was similar post-intervention (34.3%, *N* = 50, *p* > 0.05). At the baseline, a high percentage of participants (60.8%, *N* = 89) reported that they would prefer to use electronic devices rather than playing outside and this was reduced following the intervention (43.9%, *N* = 65, *p* < 0.05). 

### 3.5. Accelerometer Data

As demonstrated in Table 1, there were no observed differences in any of the parameters assessed via the accelerometer. The sedentary time (mean difference 0.2 ± 0.4%, *p* > 0.05), light activity (mean difference 0.9 ± 0.1%, *p* > 0.05), moderate activity (mean difference 0.2 ± 2.0%, *p* > 0.05) and vigorous activity (mean difference 1.0 ± 1.5%, *p* > 0.05) were all similar post-intervention when compared with the pre-intervention values. Physically active minutes were expressed relative to school days and weekends. During school days (Monday–Friday) there was no change in sedentary activity (pre: 435.3 ± 36.2 min vs. post: 403.6 ± 48.3 min, respectively, *p* > 0.05). The total light activity was reduced following the intervention (pre: 120.1 ± 10.7 min vs. post: 98.5 ± 15.7 min, respectively, *p* < 0.05) and the time spent achieving moderate activity was increased (pre: 173.6 ± 12.4 min vs. post: 198.5 ± 15.5 min, respectively, *p* < 0.05). Vigorous activity was reduced following the intervention (pre: 21.9 ± 4.4 min and post: 25.9 ± 4.5 min, respectively, *p* < 0.05). The average time being spent sedentary at weekends was reduced (pre: 539.7 ± 97.3 min vs. post: 449.8 ± 106.7 min, mean reduction 109.9 ± 9.4 min, *p* < 0.05). Despite a reduction in sedentary time and reported increases for each intensity, there were no significant changes in light activity patterns (pre: 104.2 ± 21.0 min and post: 131.8 ± 16.2 min, *p* > 0.05), moderate activity levels (pre: 138.5 ± 42.9 min and post: 169.6 ± 45.1 min, *p* > 0.05) and vigorous activity (pre: 14.6 ± 16.1 min and post: 24.8 ± 22.8 min, *p* > 0.05) during weekend days (Saturday and Sunday). When comparing weekday to weekend days, the only difference that was observed was an increased sedentary time (weekdays 539.7 ± 97.3 vs. weekend days 429.8 ± 106.7, *p* < 0.05), which was not changed post-intervention (pre: 403.6 ± 48.3 min vs. post: 435.3 ± 36.2, *p* > 0.05). 

## 4. Discussion

Although many interventions exist to enhance PA and related factors, a limitation of previous approaches is the lack of theoretical underpinning. This is coupled with a focus on pre-post-assessment and little consideration of the process evaluation in terms of how interventions run. The present study addressed these issues by designing and developing a 12-week multi-component school-based physical activity intervention that was designed using the theoretical basis and concepts of the COM-B model to affect physical activity (primary outcome). The intervention presented here was developed alongside known determinants of physical activity (e.g., sedentary behaviours, use of technologies, healthy eating, sleep and screen time) and extended the extant literature relating to multi-component physical activity interventions. The primary finding here showed that whilst the intervention was ineffective at increasing the total physical activity (primary outcome), there were changes in the broader determinants of positive health behaviours (consumption of healthy foods and sleep) but there was no change in the consumption of unhealthy snacks and contact with technological devices.

The development of health and associated social and economic benefits of physical activity is extremely strong, with increasing physical activity recognised as an international priority by the Chief Medical Officer and the recently revised physical activity guidelines [1]. Despite the need, there is a dearth in the supporting literature demonstrating impactful and lasting changes leading to population-level improvements in health and other important outcomes [27]. Whilst the intervention here was not successful at increasing physical activity, there remains a need for an effective intervention. Multi-component approaches that increase the knowledge and understanding of physical activity and healthy lifestyles remain dominated by studies on short-term, small-scale, individual-level interventions that are difficult to replicate on a broader scale [28]. The intervention here considered the important determinants of physical activity and the broader determinants of maintaining a healthy lifestyle using educational and interactive resources. Whilst the observed changes in activity levels were small, the increased knowledge and awareness could have an important long-term impact and requires further investigation and should be investigated using longitudinal approaches. 

The efficacy of physical activity interventions has also been criticised due to a lack of theoretical consideration that underpins and applies important concepts in the design and implementation stages [29]. The intervention used here was developed using the recommendations of the COM-B model proposed by Michie et al. [18]. This model provides a framework that links specific behaviour change techniques derived from the Behaviour Change Wheel and Taxonomy of Behaviour Change Techniques and has previously demonstrated an effective change in physical activity levels of primary school children [30]. Despite the present intervention being underpinned by theoretical concepts, the intervention was ineffective at changing physical activity levels in primary school children. A recent review [31] highlighted that interventions targeting an improved physical activity in children and adolescents are guilty of focusing too much attention on the complex behavioural constructs or social-ecological frameworks or a combination of both, thus leading to minimal or no improvements in the physical activity levels of young people. The authors proposed that the limited impact of interventions was likely caused because the fundamental mechanisms of change were overlooked and/or not considered in their entirety. They concluded that experimental interventions aimed at increasing physical activity levels in children should facilitate an increased access to physical activities for children to be active (i.e., facilitate access to new skills and environments), extend the existing physical activity by increasing the time allocated for opportunities and finally increase exposure to existing physical activities through strategies designed to increase physical activity above routine practice [31]. Whilst the present intervention provided bespoke opportunities to be active outside of curricular activities, there was little opportunity to expand and enhance activities as sessions were delivered in schools and dependent upon available equipment, resources and expertise as well as external and environmental influencers (such as time of year and weather conditions), which played a part in dictating the content of the intervention. In the development of future interventions, there is a need to consider and incorporate additional thinking in relation to the available equipment, resources and facilities in the development of the activities that can be used to support the widespread delivery and the scaling of future interventions. 

The variance in the observed changes observed here was indicative of previous research in this area and was likely caused by a lack of consideration of the broader relationships and influencers. In the pursuit of increased physical activity and broader health behaviours, interventions must consider the direct influencing agents and the ways that these function within a complex system [32]. System thinking and mapping literature emphasises the need to identify and study the whole system and the dynamic interrelations [33] rather than singularly identifying individual elements in isolation. Although the intervention here aimed to improve physical activity through increased knowledge and understanding, it was apparent that the design and implementation of the intervention did not consider the wider impacts and the relations between the target audience (school children) and the primary influencers and therefore adopting a broader and system thinking approach within the design process may lead to a more nuanced understanding of the impacts that interventions might have on physical activity [34]. An increased effectiveness could also be obtained, combining system thinking approaches with asset mapping, which has been demonstrated as an alternative approach to increasing health promotion outcomes [35]. Although asset mapping does not build capacity, it does represent an economical approach for identifying resources that increase capabilities and opportunities for physical activity [36]. When combined with contemporary systems, thinking approaches may lead to an increased effectiveness of interventions and a realisation of the broad impact potential in school and community settings. 

A further consideration, highlighted by recent literature, is the need to address the capability of school children to produce a competent fundamental movement skills profile [37]. Whilst there is a need to have interventions that are underpinned with a theoretical framework and contain engaging activities, there is also a need to consider the competency profile of the participant’s fundamental movement skills to ensure that the participants can complete and engage with the planned activities. Furthermore, Duncan et al. [37], demonstrate low competency when conducting fundamental movement skills. Future interventions should therefore consider approaches that test and increase the movement proficiency of participants. The considerations of movement proficiency were not contained in the current intervention and whilst not definitive it is plausible that this contributed to the minimal increases in the total physical activity. Previously, Bryant et al. [38] demonstrated a low fundamental movement skill proficiency in British children and other work outside of the UK has demonstrated similarly low results when using objective measurement criteria [39]. Duncan et al. [37] were first to provide proficiency-level data indicating that functional movement proficiency is very poor in British schoolchildren when considered with the fundamental skills specified by the National Curriculum for physical education in England. This highlights the need to address the capabilities of school children and enhance the mastery of basic skills to enable the development of more complex skills during the later stages of life, leading to an enhanced enjoyment and lifelong participation in sport and physical activities [40].

## 5. Conclusions

Despite an increased knowledge and understanding of physical activity requirements, healthy lifestyles were increased yet total physical activity was unchanged in this intervention. Whilst the use of theoretical approaches is needed to increase activity levels, future work in this area must also consider the complex systems and agents by which physical activity is influenced. 

## Figures and Tables

**Table 1:** Physical activity percentage breakdown according to intensity pre- and post-intervention using a wrist-worn accelerometer (*N* = 11).

	Pre (%)	Post (%)	*p* Value
Sedentary Activity	59.8 ± 3.0	59.6 ± 3.4	0.643
Light Activity	15.2 ± 1.0	14.3 ± 0.9	0.150
Moderate Activity	22.4 ± 1.9	22.6 ± 3.9	0.587
Vigorous Activity	2.6 ± 0.8	3.6 ± 2.3	0.049

All values are mean ± standard deviation.

## Data Availability

Data are available upon request.
